# Total Knee Arthroplasty in the Presence of Congenital Heart Disease: An Under-Recognized Risk

**DOI:** 10.1016/j.artd.2026.102063

**Published:** 2026-06-08

**Authors:** Seyed Morteza Kazemi, Ava Parvandi, Amir-Mohammad Asgari, Pooya Hosseini-Monfared, Donya Rezazadeh Eidghahi, Sepehr Rezazadeh Eidghahi, Mahdi Mohammaditabar, Shayan Amiri, Alireza Mirahmadi

**Affiliations:** a Bone Joint and Related Tissues Research Center, Akhtar Orthopedic Hospital, Shahid Beheshti University of Medical Sciences, Tehran, Iran; b Department of Orthopedics, Bone and Joint Reconstruction Research Center, School of Medicine, Iran University of Medical Sciences, Tehran

**Keywords:** Total knee arthroplasty, Congenital heart disease, Patent foramen ovale, Paradoxical embolism, Embolic stroke, Transesophageal echocardiography

## Abstract

**Background:**

Adults with congenital heart disease (CHD) increasingly present for noncardiac surgery. Total knee arthroplasty (TKA) induces significant hemodynamic and thromboembolic stress and, in patients with intracardiac shunts such as patent foramen ovale (PFO) or atrial septal defect (ASD), may precipitate paradoxical embolism and other under-recognized complications.

**Methods:**

A systematic review was conducted in accordance with the Preferred Reporting Items for Systematic Reviews and Meta-Analyses guidelines. PubMed, Scopus, Embase, and Web of Science were searched from inception to December 2024. Peer-reviewed original studies reporting outcomes of TKA in patients with CHD were eligible. Two reviewers independently screened studies, extracted data, and appraised methodological quality using the Joanna Briggs Institute tools. Given the heterogeneity and predominance of case-based evidence, findings were synthesized narratively.

**Results:**

Eleven case reports (1996-2023) involving 11 patients met the inclusion criteria. Most patients were female (72.7%), with a mean age of 67.3 years (range, 52-84). PFO was the most frequent lesion (81.8%), followed by ASD (18.2%). Symptom onset was commonly early: 36.4% occurred perioperatively and 36.4% within the first 2 postoperative days. Neurological manifestations predominated (72.7%), with respiratory symptoms (27.3%) and limb ischemia (9.1%) less frequent. Transesophageal echocardiography confirmed the diagnosis in 72.7% of cases. Paradoxical cerebral embolism/stroke was the principal complication; pulmonary embolism (18.2%) and venous thromboembolism (9.1%) were also reported.

**Conclusions:**

TKA in patients with CHD, particularly PFO or ASD, may be complicated by early paradoxical embolic events. New neurological deficits or unexplained hypoxemia warrant urgent evaluation for intracardiac shunting. Higher-quality studies are needed to guide screening and prevention strategies.

## Introduction

Congenital heart disease (CHD) encompasses a spectrum of abnormalities, from simple lesions such as atrial septal defect (ASD), ventricular septal defect, and patent foramen ovale (PFO), to complex cyanotic malformations that lead to chronic hypoxemia, pulmonary hypertension, arrhythmias, and progressive heart failure [1]. Advances in pediatric cardiology, cardiac surgery, and long-term medical management have substantially improved survival, resulting in an increasing number of adults with CHD presenting for noncardiac surgical procedures [2].

Total knee arthroplasty (TKA) is a widely performed major orthopaedic procedure that effectively treats end-stage knee osteoarthritis and other degenerative joint disorders [3]. Despite its overall safety, TKA imposes significant physiological stress and carries perioperative and postoperative risks, especially in patients with underlying cardiovascular disease [4]. Adults with CHD represent a uniquely vulnerable subgroup because of abnormal cardiac anatomy, residual intracardiac shunts, impaired cardiopulmonary reserve, and altered hemodynamics [5].

Venous thromboembolism (VTE) is among the most serious complications following total joint arthroplasty [6]. Postoperative immobility and the inflammatory response to surgery increase the risk of deep vein thrombosis and pulmonary embolism (PE) [7]. In addition to thromboembolic complications, other systemic risks such as acute kidney injury and metabolic stress have been increasingly recognized, particularly in higher-risk procedures such as simultaneous bilateral TKA [8,9]. In patients with intracardiac communications, particularly PFO, embolic material may cross from the venous to the arterial circulation, potentially leading to ischemic stroke or other systemic embolic events, a mechanism often referred to as paradoxical embolism [10,11].

In patients with CHD, perioperative hemodynamic changes can elevate right-sided cardiac pressures and promote right-to-left shunting, leading to hypoxemia, arrhythmias, and hemodynamic instability [12,13]. Rare but serious complications, such as platypnoea-orthodeoxia syndrome, may also occur, and delayed recognition of these conditions is associated with worse outcomes [14]. Additionally, unrecognized intracardiac shunts can predispose patients to neurological events, including cryptogenic ischemic stroke and transient ischemic attack, during the postoperative period following orthopaedic surgery [15].

Although similar mechanisms have been described in hip arthroplasty [[16], [17], [18]], data specific to TKA in adults with CHD remain limited. With the rising prevalence of both CHD survival into adulthood and TKA utilization, this patient population is increasingly encountered in clinical practice. Improved awareness of CHD-related perioperative complications is essential to guide risk assessment, optimize perioperative management, and prevent adverse outcomes. This review examines the interaction between CHD and TKA, aiming to improve recognition and management in this high-risk population.

## Material and methods

This systematic review was conducted in accordance with the Preferred Reporting Items for Systematic Reviews and Meta-Analyses (PRISMA) guidelines. The review protocol was registered in the International Prospective Register of Systematic Reviews (PROSPERO) database (CRD420261323718). The objective of the review was to evaluate perioperative and postoperative complications of TKA in patients with CHD, with particular attention to embolic, neurological, and hemodynamic events.

### Search strategy

A comprehensive literature search was performed using PubMed, Scopus, Embase, and Web of Science from database inception to December 2024. The search strategy combined Medical Subject Headings (MeSH) terms and free-text keywords related to TKA and CHD. Search terms included combinations of (“total knee arthroplasty” OR “knee replacement” OR “TKA”) AND (“congenital heart disease” OR “patent foramen ovale” OR “atrial septal defect” OR “intracardiac shunt” OR “paradoxical embolism”). Reference lists of included articles and relevant reviews were manually screened to identify additional eligible studies.

### Eligibility criteria

Peer-reviewed original studies reporting outcomes of TKA in patients with CHD were eligible for inclusion. Case reports, case series, and observational studies were included due to the rarity of the condition and the limited availability of large-cohort studies. Reviews, editorials, commentaries, conference abstracts, and non–peer-reviewed articles were excluded. No restrictions were placed on language or year of publication. Only studies with accessible full texts were included to allow a comprehensive assessment of clinical presentation, diagnostic evaluation, and outcomes.

### Study selection

**Figure 1:**
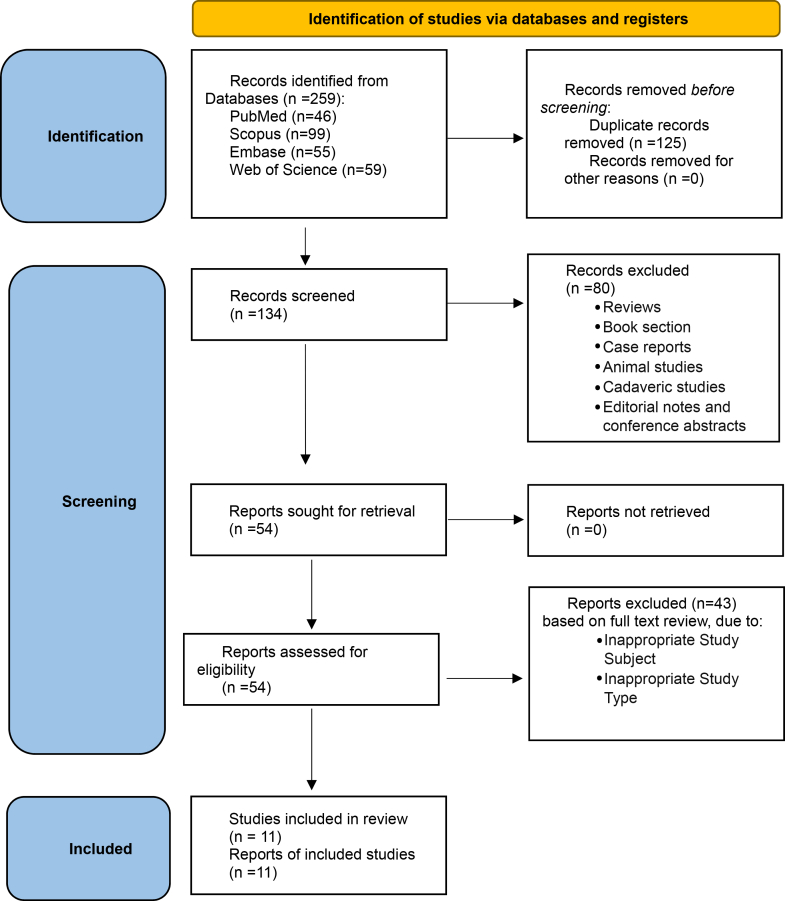
Study selection based on the Preferred Reporting Items for Systematic Reviews and Meta-Analyses (PRISMA) 2018 statement.

Two reviewers independently screened titles and abstracts to identify potentially relevant studies. Full-text articles were obtained for all studies that met the inclusion criteria or for which eligibility was uncertain. Final study inclusion was determined through independent full-text review, with discrepancies resolved by consensus or consultation with a third reviewer. The study selection process is summarized using a PRISMA flow diagram (Fig. 1).

### Quality assessment

Methodological quality was assessed using the Joanna Briggs Institute Critical Appraisal Checklist, selected according to study design. Case reports and case series were evaluated across domains, including clarity of patient demographics, clinical history, diagnostic methods, intervention description, outcome reporting, and identification of adverse events. Quality assessment was performed independently by 2 reviewers, with disagreements resolved through discussion or adjudication by a third reviewer. Overall study quality was categorized as high, moderate, or low, acknowledging the inherent limitations of case-based evidence (Table 1).

**Table 1:** Quality assessment of the included studies.

JBI Critical appraisal checklist	Weiss et al. [14]	Ogino et al. [15]	Yeon et al. [16]	Tangsataphorn et al. [19]	Calcaterra et al. [18]	Hill et al. [17]	Bruce-Br et al. [20]	Cho et al. [21]	Almufti et al. [22]	Mojaddedi et al. [11]
Were patient’s demographic characteristics clearly described?	Yes	Yes	Yes	Yes	Yes	Yes	Yes	Yes	Yes	Yes
Was the patient’s history clearly described and presented as a timeline?	Yes	Yes	Yes	Yes	Unclear	Unclear	Yes	Yes	Yes	Yes
Was the current clinical condition of the patient on presentation clearly described?	Yes	Yes	Yes	Yes	Yes	Unclear	Yes	Yes	Yes	Yes
Were diagnostic tests or assessment methods and the results clearly described?	Yes	Yes	Yes	Yes	Yes	Yes	Yes	Yes	Yes	Yes
Was the intervention(s) or treatment procedure(s) clearly described?	Unclear	Unclear	Yes	Unclear	Unclear	Unclear	Yes	Yes	Unclear	Yes
Was the postintervention clinical condition clearly described?	Yes	Yes	Yes	Yes	Unclear	Unclear	Yes	Yes	Yes	Yes
Were adverse events (harms) or unanticipated events identified and described?	Yes	Yes	Yes	Yes	Yes	Yes	Yes	Yes	Yes	Yes
Does the case report provide takeaway lessons?	Yes	Yes	Yes	Yes	Unclear	Yes	Yes	Yes	Yes	Yes

### Data extraction and synthesis

Data were extracted using a standardized data collection form that captured study characteristics, patient demographics, type of CHD, perioperative findings, clinical presentation, diagnostic modalities, management strategies, and outcomes. Data extraction was conducted independently by 2 reviewers to minimize bias, with discrepancies resolved through discussion or third-party review. Owing to heterogeneity in study design, patient characteristics, and reported outcomes, a narrative synthesis was undertaken. Findings were organized thematically to identify recurring patterns, key complications, and clinically relevant trends across the included studies.

## Results

A total of 10 case reports published between 1996 and 2023 were included, comprising 11 patients. Most patients were female (8/11, 72.7%), and the age ranged from 52 to 84 years (mean 67.3 years; median 65 years). Body mass index was reported in 3 cases (24.3-42.6 kg/m^2^) (Table 2)

**Table 2:** Characteristics of the included studies.

First author	Year	Design	N	Sex	Age (y)	BMI	Key comorbidities	Shunt/defect	Anesthesia	Symptom onset after surgery	Postop presentation	Main diagnostic tool	Key imaging/echo findings	Complications
Weiss et al. [14]	1996	Case report	1	F	84	NR	HTN, rheumatoid arthritis	LVH + PFO	General	After tourniquet deflation; deterioration described on POD3	Severe depressed consciousness/neurologic decline	TEE	Preop: LVH; postop: severe neurologic depression described	Paradoxic cerebral embolism
Ogino et al. [15]	1999	Case report	1	F	76	24.9	NM	Small ASD (<3 mm), L→R shunt	Spinal	∼20 min after bone-cement implantation	L facial palsy; L arm paresis; global aphasia	TTE	ASD with shunt; acute neurologic deficits temporally related to cementing	Cerebral infarction
Yeon et al. [16]	2003	Case report	1	M	65	42.6	LBBB	PFO	Epidural	After tourniquet deflation	Dysarthria; cranial nerve findings	TEE	TEE: functional R→L shunt without provocative maneuvers	Paradoxic cerebral embolism
Yeon et al. [16]	2003	Case report	1	F	64	24.3	End-stage OA	Aneurysmal IAS + small secundum ASD with R→L flow	Epidural	PACU	Dysarthria; facial droop; mild hand weakness; lethargy	TEE	Brain CT: multifocal embolic infarcts; TEE: ASD/IAS aneurysm with visible R→L flow	Paradoxic cerebral embolism
Tangsataphorn et al. [19]	2009	Case report	1	F	62	NM	Hypertension	PFO	NM	ICU course over days (leg ischemia day 2; later neuro/visual symptoms)	Dyspnea/drowsiness; acute limb ischemia; later visual field loss	TEE	Reported diagnoses: pulmonary embolus; acute popliteal occlusion; occipital infarction	Paradoxical embolism, PE reported + arterial occlusion
Calcaterra et al. [18]	2010	Case report	1	M	65	NM	NM	Dilated RV + PFO	NM	∼3 wk	Lightheadedness; acute RUE weakness; AMS/seizure	TEE	Imaging: bilateral PE; acute right subclavian thromboembolism/occlusion; TEE: PFO	PE reported
Hill et al. [17]	2012	Case report	1	F	52	NM	NM	PFO	NM	NM	NM	TEE	NM	Paradoxical cerebral embolism
Bruce-Br et al. [20]	2013	Case report	1	M	65	NM	Bilateral knee osteoarthritis; ischemic heart disease with prior MI; triple CABG; diet-controlled T2DM	Mildly dilated left atrium + PFO	Spinal	Morning after surgery	Right facial droop; right upper limb weakness; dysarthria (slurred speech)	TTE	Echo: mildly dilated left atrium; PFO	Cerebrovascular infarction
Cho et al. [21]	2015	Case report	1	F	64	NM	HTN, diabetes	PFO	NM	Second wk after surgery	Dyspnea; brain infarction	TEE	Echo: mobile RA thrombi; RV enlargement, severe pulmonary hypertension, D-shaped LV	VTE reported
Almufti et al. [22]	2019	Case report	1	F	82	NM	Prior MI (10y), HTN, ascending aortic aneurysm (∼5 cm)	PFO	NM	First day after surgery	R arm weakness; L homonymous hemianopia; hypoxia	TTE	TTE: preserved LV function (EF >50%), mild aortic/mitral degenerative changes; brain MRI/CT: multifocal acute infarcts (embolic pattern); CTPA: no PE, RV strain/atelectasis	Platypnoea–orthodeoxia syndrome
Mojaddedi et al. [11]	2023	Case report	1	F	61	NM	Rheumatoid arthritis; hysterectomy; T2DM (controlled); HTN	PFO with large R→L shunt	NM	Postop day 2	Acute R-sided hemiparesis	TEE	TEE: large R→L shunt via PFO; pulmonary embolus listed	Cardioembolic stroke

CABG, coronary artery bypass grafting; T2DM, type 2 diabetes; PACU, post-anesthesia care unit; ICU, intensive care unit; RUE, right upper extremity; F/M, female/male; AMS, altered mental status; RA, right atrium; EF, ejection fraction; LVH, left ventricular hypertrophy; CT, computer tomography; POD3, post operation day 3; OA, osteoarthritis; HTN, hypertension; MI, myocardial infarction; IAS, interatrial septum; LBBB, left bundle branch block; TTE/TEE, transthoracic/transesophageal echocardiography; CTPA, CT pulmonary angiography; BMI, body mass index; MRI; magnetic resonance imaging; RV/LV, right/left ventricle; NM, not mentioned.

A PFO was the predominant intracardiac defect (9/11, 81.8%), whereas ASD-related lesions were reported in 2/11 (18.2%) cases (including a small secundum ASD with aneurysmal interatrial septum and right-to-left flow). Anesthesia type was documented in 5 reports (2 spinal, 2 epidural, and 1 general), but not in the remaining cases.

Symptom onset was typically early. Four patients (36.4%) developed symptoms in the immediate perioperative period (after tourniquet deflation, in the postanesthesia care unit, or within 20 minutes of cement implantation), and another 4 (36.4%) presented within the first 2 postoperative days. One report described onset in the second postoperative week, and 1 described onset approximately 3 weeks postoperatively; timing was not reported in 1 case.

Neurologic manifestations were the dominant clinical presentation, reported in 8/11 patients (72.7%), including dysarthria, facial droop, hemiparesis/weakness, aphasia, altered mental status, seizure, or visual field loss. Respiratory symptoms (dyspnea/shortness of breath or hypoxia) were reported in 3/11 cases (27.3%), and acute limb ischemia/arterial occlusion occurred in 1 case (9.1%). Transesophageal echocardiography was the primary diagnostic modality in most cases (8/11, 72.7%), with transthoracic echocardiography used in 3/11 (27.3%).

Reported complications were predominantly paradoxical cerebral embolism/stroke (including cerebral infarction and cardioembolic stroke). PE was explicitly documented in 2 cases (18.2%), and VTE was reported in 1 case (9.1%). Outcome and mortality data were inconsistently reported across case reports and were not suitable for pooled analysis.

## Discussion

Demographically, the reviewed cases predominantly involved older adults, with reported ages ranging from the early 50s to the mid-80s. A female predominance was observed across the cases. Many patients had cardiovascular comorbidities such as hypertension, prior myocardial infarction, coronary artery bypass surgery, diabetes mellitus, or obesity; however, several patients had unremarkable preoperative cardiac assessments and no known neurological disease. Notably, none of the reported complications were related to prosthetic failure, and revision arthroplasty was not required in any case. A variety of anesthetic techniques were used across the studies, including spinal, epidural, and combined regional anesthesia. Some authors noted that tourniquet release and limb reperfusion were temporally associated with hemodynamic changes favoring right-to-left shunting, including increased pulmonary vascular resistance and elevated right atrial pressure [21,19].

Management strategies varied depending on clinical severity and institutional practice. Most patients were treated with therapeutic anticoagulation and supportive neurological care [11,13,21,[22], [23], [24], [25]]. In selected cases, percutaneous PFO closure was performed following the index embolic event, with favorable outcomes and no recurrence during follow-up [13,25]. Yeon et al. [22] also described the novel use of temporary intraoperative balloon occlusion of a PFO to enable safe completion of subsequent knee arthroplasty, highlighting a potential preventive strategy for carefully selected high-risk patients. Despite these interventions, mortality was reported in 1 case, emphasizing that outcomes may be poor when diagnosis is delayed or the embolic burden is extensive [21]. Across the analyzed studies, neurological complications were the most common and clinically significant postoperative presentation. Multiple authors reported acute-onset focal neurological deficits occurring within hours to days after TKA, including hemiparesis, facial droop, dysarthria, aphasia, seizures, altered mental status, and reduced consciousness [11,13,21,[22], [23], [24], [25]].

PFO is a common, often clinically silent remnant of fetal circulation that may become pathologically relevant under specific physiological stressors [13]. The reviewed literature demonstrates that TKA can act as a precipitating event for paradoxical embolism in patients with previously undiagnosed PFO, leading to severe neurological and systemic complications [11,21,[23], [24], [25]]. Although paradoxical embolism remains rare, its consequences following TKA are potentially catastrophic and frequently represent the first clinical manifestation of an underlying intracardiac shunt [11,13,21,22,26].

Yeon et al. [22] described 2 patients who developed paradoxical cerebral embolism following cemented TKA, presenting with dysarthria, facial weakness, and limb paresis. In both cases, diffusion-weighted magnetic resonance imaging confirmed acute embolic infarcts, and transesophageal echocardiography revealed right-to-left shunting through a PFO or ASD [22]. Similar neurological presentations were reported by Hill et al. [11], Calcaterra et al. [23], and Tangsataphorn et al. [25], in which patients developed postoperative stroke syndromes without prior cerebrovascular disease, ultimately attributed to paradoxical embolism through an unrecognized PFO.

Several reports highlighted the severity of neurological injury associated with this mechanism [11,21,22,23,25]. Weiss et al. [21] described a fatal paradoxical cerebral embolization during bilateral knee arthroplasty, underscoring the potential lethality of this complication. Other cases documented extensive multifocal cerebral infarction, obtundation, absent brainstem reflexes, and decerebrate posturing, consistent with widespread embolic burden [21,22,24]. In contrast, patients with smaller embolic events often demonstrated partial or complete neurological recovery following anticoagulation and supportive management, indicating a broad clinical spectrum [11,13,22,23,25].

In addition to cerebral embolism, systemic arterial and venous thromboembolic complications were frequently observed. Cho et al. [24] reported a complex postoperative course involving deep vein thrombosis, PE, right atrial thrombi, and embolic stroke in a single patient with PFO following TKA. Similar patterns were described in other cases, where PE and hypoxemia preceded or accompanied neurological deterioration. One patient developed acute arterial insufficiency of the lower limb requiring embolectomy, followed by subsequent cerebral infarction, further illustrating that paradoxical emboli may affect extracranial vascular territories [25].

Platypnoea-orthodeoxia syndrome represents a distinctive but under-recognized manifestation in this population. Almufti et al. [26] described a patient who developed positional hypoxemia following TKA, with posture-dependent oxygen desaturation in the upright position and partial improvement when supine. Extensive investigation ultimately demonstrated PFO-related right-to-left shunting as the underlying mechanism. This presentation highlights the importance of considering intracardiac shunt physiology in patients with unexplained postoperative hypoxemia, particularly when symptoms are posture-dependent.

Adults with CHD undergoing TKA have both physiological and psychosocial vulnerability. Depression is more prevalent in CHD (9%-30%) and reflects lifelong disease burden and functional limitation [27]. It has been shown that perioperative depression use has also been associated with increased opioid-related adverse outcomes after arthroplasty [28]. These factors may influence perioperative recovery and pain perception. Opioid analgesics can cause respiratory depression, hypoventilation, and hypercapnia, increasing pulmonary vascular resistance and right-sided pressures [29]. In patients with intracardiac shunts, this may worsen right-to-left shunting and hypoxemia, while inadequate analgesia may trigger sympathetic instability. Together, these findings highlight combined physiological and psychological vulnerability and support a multidisciplinary perioperative approach.

Overall, the reviewed literature identifies PFO-related paradoxical embolism as a rare but serious complication of TKA. Neurological deficits, particularly embolic stroke, were the most common and often the presenting manifestation, frequently occurring in patients without prior cerebrovascular disease or known intracardiac shunts. Given the high prevalence of undiagnosed PFO in the general population and the increasing volume of TKA procedures, heightened clinical awareness is essential. Early recognition of postoperative neurological symptoms, unexplained hypoxemia, or systemic embolic phenomena should prompt consideration of paradoxical embolism and targeted diagnostic evaluation. Further studies are required to clarify the role of preoperative screening, perioperative risk stratification, and preventive strategies in this vulnerable patient population.

## Conclusions

Paradoxical embolic events after orthopaedic surgery are rare but potentially catastrophic and, in the available case-based literature, occur predominantly in older patients with an underlying interatrial communication, most commonly a PFO. Presentations are typically early and are driven by acute neurologic deficits, sometimes accompanied by hypoxemia or venous thromboembolic features. Because transesophageal echocardiography most consistently confirmed right-to-left shunting in reported cases, a high index of suspicion and prompt cardiopulmonary evaluation are warranted when new neurologic symptoms or unexplained hypoxia develop after surgery, particularly around tourniquet deflation or cement implantation. However, evidence is limited to case reports with incomplete and heterogeneous reporting, preventing robust risk estimation and outcome synthesis. Future prospective studies and standardized reporting are needed to better define perioperative predictors, clarify the relationship with VTE/PE, and inform prevention and management strategies in patients with known or suspected interatrial shunts.

## CRediT authorship contribution statement

**Seyed Morteza Kazemi:** Writing – review & editing, Writing – original draft, Supervision. **Ava Parvandi:** Writing – review & editing, Writing – original draft, Visualization, Supervision, Methodology, Formal analysis, Data curation. **Amir-Mohammad Asgari:** Methodology, Formal analysis, Data curation. **Pooya Hosseini-Monfared:** Writing – original draft, Project administration, Data curation, Conceptualization. **Donya Rezazadeh Eidghahi:** Writing – review & editing, Writing – original draft, Visualization. **Sepehr Rezazadeh Eidghahi:** Writing – review & editing, Writing – original draft, Data curation. **Mahdi Mohammaditabar:** Methodology, Formal analysis. **Shayan Amiri:** Visualization, Investigation. **Alireza Mirahmadi:** Writing – review & editing, Writing – original draft, Visualization, Validation, Methodology, Formal analysis, Data curation, Conceptualization.

## Conflicts of interest

The authors declare there are no conflicts of interest.

For full disclosure statements refer to https://doi.org/10.1016/j.artd.2026.102063.

## Appendix A Supplementary data

Conflict of Interest Statement for Asgari

Conflict of Interest Statement for Mirahmadi

Conflict of Interest Statement for Parvandi

Conflict of Interest Statement for Eidghahi

Conflict of Interest Statement for Mohammaditabar

Conflict of Interest Statement for Monfared

Conflict of Interest Statement for Amiri

Conflict of Interest Statement for Kazemi

Conflict of Interest Statement for Eidghahi
