# Supplementary material for: Total Knee Arthroplasty in the Presence of Congenital Heart Disease: An Under-Recognized Risk
Source: Arthroplast Today. 2026 Jun 8;39:102063. doi: 10.1016/j.artd.2026.102063 (PMC13264072; doi:10.1016/j.artd.2026.102063)
Supplement: Conflict of Interest Statement for Eidghahi [file mmc4.docx]

# CONFLICT OF INTEREST STATEMENT

***American Association of Hip and Knee Surgeons***

(Adopted from the American Academy of Orthopaedic Surgeons disclosure statement)

The following form **must be filled out completely and submitted by each author (example, 6 authors, 6 forms).**

**All items require a response. If there is no relevant disclosure for a given item, enter "*None*.”**

**Total Knee Arthroplasty in the Presence of Congenital Heart Disease: An Under-Recognized Risk**

Manuscript Title

1. Royalties from a company or supplier (The following conflicts were disclosed): None

2. Speakers bureau/paid presentations for a company or supplier (The following conflicts were disclosed) : None

3A. Paid employee for a company or supplier (The following conflicts were disclosed) : None

3B. Paid consultant for a company or supplier (The following conflicts were disclosed) : None

3C. Unpaid consultants for a company or supplier (The following conflicts were disclosed) : None

4. Stock or stock options in a company or supplier (The following conflicts were disclosed) : None

5. Research support from a company or supplier as a Principal Investigator (The following conflicts were disclosed) : None

6. Other financial or material support from a company or supplier (The following conflicts were disclosed) : None

7. Royalties, financial or material support from publishers (The following conflicts were disclosed) : None

8. Medical/Orthopaedic publications editorial/governing board (The following conflicts were disclosed) : None

9. Board member/committee appointments for a society (The following conflicts were disclosed) : None

**Each author must sign AND print or type his/her name, date and submit a separate form**

In addition, one BLINDED Conflict of Interest form (no author names used) should be submitted per manuscript with all author disclosures.

Author Name **Donya Rezazadeh Eidghahi** Author Signature **Donya Rezazadeh Eidghahi** Date 22 february 2026
